# Deep learning models for screening of high myopia using optical coherence tomography

**DOI:** 10.1038/s41598-021-00622-x

**Published:** 2021-11-04

**Authors:** Kyung Jun Choi, Jung Eun Choi, Hyeon Cheol Roh, Jun Soo Eun, Jong Min Kim, Yong Kyun Shin, Min Chae Kang, Joon Kyo Chung, Chaeyeon Lee, Dongyoung Lee, Se Woong Kang, Baek Hwan Cho, Sang Jin Kim

**Affiliations:** 1 Department of Ophthalmology, Samsung Medical Center, Sungkyunkwan University School of Medicine, #81 Irwon-ro, Gangnam-gu, Seoul, 06351 Republic of Korea; 2 Medical AI Research Center, Samsung Medical Center, #81 Irwon-ro, Gangnam-gu, Seoul, 06351 Republic of Korea; 3 Department of Ophthalmology, Samsung Changwon Hospital, Sungkyunkwan University School of Medicine, Changwon, Republic of Korea; 4 Department of Ophthalmology, Gil Medical Center, Gachon University, Incheon, Republic of Korea; 5 Nune Eye Hospital, Seoul, Republic of Korea; 6 Department of Medical Device Management and Research, SAIHST, Sungkyunkwan University, Seoul, 06351 Republic of Korea

**Keywords:** Diseases, Medical research

## Abstract

This study aimed to validate and evaluate deep learning (DL) models for screening of high myopia using spectral-domain optical coherence tomography (OCT). This retrospective cross-sectional study included 690 eyes in 492 patients with OCT images and axial length measurement. Eyes were divided into three groups based on axial length: a “normal group,” a “high myopia group,” and an “other retinal disease” group. The researchers trained and validated three DL models to classify the three groups based on horizontal and vertical OCT images of the 600 eyes. For evaluation, OCT images of 90 eyes were used. Diagnostic agreements of human doctors and DL models were analyzed. The area under the receiver operating characteristic curve of the three DL models was evaluated. Absolute agreement of retina specialists was 99.11% (range: 97.78–100%). Absolute agreement of the DL models with multiple-column model was 100.0% (ResNet 50), 90.0% (Inception V3), and 72.22% (VGG 16). Areas under the receiver operating characteristic curves of the DL models with multiple-column model were 0.99 (ResNet 50), 0.97 (Inception V3), and 0.86 (VGG 16). The DL model based on ResNet 50 showed comparable diagnostic performance with retinal specialists. The DL model using OCT images demonstrated reliable diagnostic performance to identify high myopia.

## Introduction

High myopia is associated with many ocular complications that can threaten vision^1–3^. Due to myopic axial elongation with subsequent pathologic changes, highly myopic eyes often show abnormal fundus findings including posterior staphyloma, lacquer crack, chorioretinal atrophy, myopic traction maculopathy, macular hole and choroidal neovascularization, etc. So, detecting high myopia is important for the proper diagnosis of various retinal conditions.

High myopia is generally defined as myopia of − 6.0 diopters or more or an axial length of 26.5 mm or more^4^. Although we can easily detect most patients with high myopia by measuring refractive error which is routinely performed in ophthalmology clinics, in patients who have undergone cataract surgery or refractive surgery such as laser vision correction, measuring refractive error gives no clue for high myopia. When fundus exam reveals characteristic findings of myopic retinal conditions, we can perform A-scan ultrasonography or partial coherence interferometry to diagnose high myopia. However, when we have no clue for high myopia on fundus exam or detailed past medical history cannot be obtained, we may not be able to detect high myopia in some patients. Moreover, A-scan ultrasonography or partial coherence interferometry are not commonly used for ophthalmologic evaluation but performed in specific situations only (e.g. for intraocular lens power calculation for cataract surgery).

On the other hand, optical coherence tomography (OCT), a non-invasive imaging technique that can visualize a cross-section of the retina, is widely used by general ophthalmologists as well as retinal specialists and OCT examination is becoming very common. OCT is an essential tool for modern ophthalmologists in diagnosing and screening retinal diseases, especially those of the macula^5,6^. OCT gives detailed information about retinal microstructure in various retinal conditions including age-related macular degeneration, diabetic retinopathy, and myopia-associated retinal diseases. Ocular changes by pathologic myopia can also be visualized on OCT images in addition to fundus photographs. Various findings of pathologic myopia have been reported in several studies^7–10^. Therefore, if we can diagnose or suspect eyes with high myopia with OCT alone in patients who underwent retinal OCT, we may be able to detect more patients with high myopia.

In recent years, attempts to apply deep learning (DL) models in the ophthalmology field have been undertaken. Studies have been conducted to diagnose and evaluate the severity of diseases, such as age-related macular degeneration, diabetic retinopathy, and retinopathy of prematurity^11–14^. There was an attempt to perform differential diagnosis using the DL model based on fundus images^15–17^. In addition, segmentation of OCT images using DL is also being actively studied for mainly age-related macular degeneration^18–20^. As such, various DL studies using OCT have been conducted especially in age-related macular degeneration but not in high myopia^18,21,22^. And, there has been no previous study on whether the DL model can diagnose or screen for high myopia by OCT imaging without measuring the axial length. It will be clinically useful to screen high myopia using OCT image-based DL models.

In this study, the researchers generated DL models for the screening of high myopia using a pair of horizontal and vertical spectral-domain OCT images. The models were validated and evaluated using our in-house dataset including OCT images of normal and other retinal conditions. The results of DL classification were then compared with classification performed by ophthalmology residents and retinal specialists.

## Methods

This retrospective study was conducted at the Department of Ophthalmology, Samsung Medical Center, Seoul, Republic of Korea. Approval for this study was obtained from the Institutional Review Board (IRB) at Samsung Medical Center, Seoul, Republic of Korea (IRB approval #2021–04-039). Due to the retrospective nature of this study, exemption from written consent was approved by the IRB, and all clinical records were de-identified for anonymity before analysis. The study adhered to the tenets of the Declaration of Helsinki.

### Dataset

The researchers retrospectively reviewed electronic medical records of patients who had undergone OCT before cataract surgery at Samsung Medical Center between July 2017 and December 2019. Before cataract surgery, both OCT and axial length measurement were performed in most patients. Patients with spectral-domain OCT (Spectralis HRA + OCT; Heidelberg Engineering, Heidelberg, Germany) images containing a pair of horizontal and vertical OCT scans of fovea-centered view (9 mm in length) were included. Defocus or complex conjugate artifact can often occur as a characteristic of high myopic eyes with long axial length and severe staphyloma^7,23^. Therefore, when severe complex conjugate artifacts occurred, diopter correction was performed before OCT examination. Patients in which the retinal layer could not be discriminated, due to very severe cataract or media opacity were excluded. In addition, patients with complex-conjugate artifacts corresponding to more than one-fifth of 9 mm scans were excluded despite diopter correction before OCT imaging. The included OCT images were taken in High Speed mode provided by the manufacturer, and 768 pixels were included in the 9 mm length, and 496 pixels were included in the 1.9 mm depth. 768 A-scans were included for each B-scan image section. To improve visualization, the value of 50 to 100 scans were averaged for each section.

Included eyes were classified clinically into three groups: normal, high myopia, and other retinal diseases. For classification, two retinal specialists reviewed and annotated each patient’s horizontal and vertical OCT images in consideration of the axial length. The three groups were classified based on the following criteria to screen accurately for highly myopic eyes and to distinguish between cases with other macular diseases and normal findings. When the axial length was 26.5 mm or longer, OCT images of the patient were annotated as “high myopia”^24^. Among them, cases with retinal diseases other than myopia related features were excluded. Non-high myopia eyes were classified in the “normal” group (when there was no abnormality in the macula on OCT) or the “others” group (when there was abnormality such as drusen, epiretinal membrane, or macular edema on OCT). Each group was set to recruit 230 eyes with 230 pairs of OCT images including training and test datasets, and the 230 images were selected randomly.

### Preprocessing and data augmentation

To analyze horizontal OCT images of right and left eyes together, OCT images of the left eyes were flipped horizontally. Data augmentation is a promising way to increase the performance of classification tasks^25^ by generating more samples from the available images^26^. Brightness control and image shift cropping were adopted for data augmentation^27^. New training images were generated from the original images using 18 brightness levels between 0.1 and 1.2 and were carefully chosen by the two retinal specialists. The researchers also cropped 20 randomly selected patches from each OCT image, representing 92% of the original image. Hence, number of training images was increased by 360 times. After data augmentation, all dataset images were resized to 318 × 512 pixels. The mean subtraction was applied such that the mean image of the training dataset was subtracted from every input image.

### CNN model architectures

The CNN-based models in this study adopted VGG 16, ResNet 50, and Inception V3 as a backbone and were initialized with ImageNet-pretrained models^28^. Single-column and multiple-column CNN models were used for classification of the OCT images.

**Figure 1:**
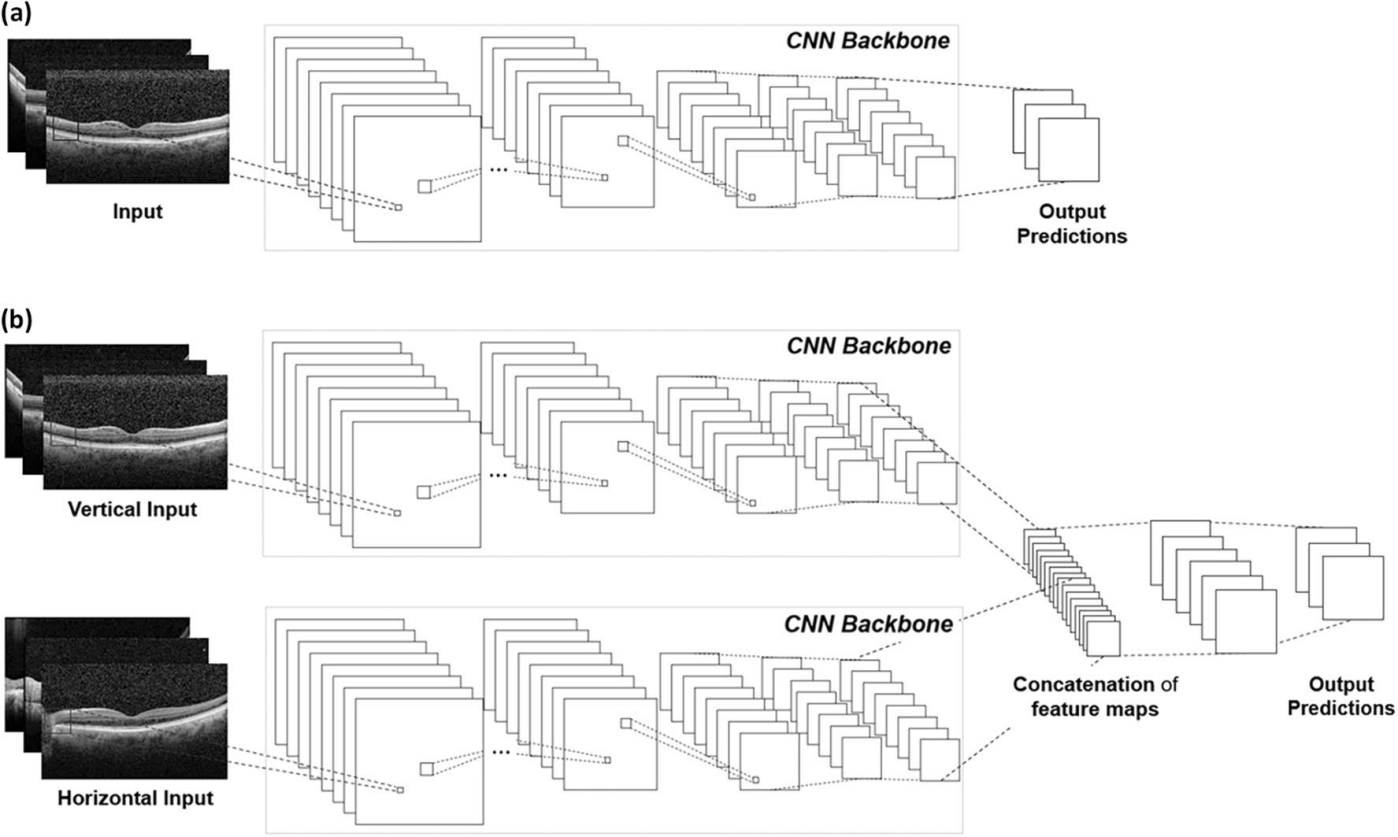
Overview of the framework. (**a**) Single-column model. (**b**) Multiple-column model, which considers vertical and horizontal OCT images simultaneously at each CNN feature extractor.

The single-column model used a single OCT image for classification, whose architecture consisted of a single CNN backbone as shown in Fig. 1a. After the original feature extracting backbone layers from VGG 16 (19 layers), Inception V3 (311 layers), and ResNet 50 (174 layers), we put a single fully-connected layer for VGG 16 and Inception V3, and 3 fully-connected layers for ResNet 50. Thus, vertical and horizontal models were trained separately for vertical and horizontal images for 20 epochs with an initial learning rate of 0.001, batch size of four, and stochastic gradient descent optimization.

Since class annotation was performed for each eye rather than each of the vertical and horizontal images separately, the multiple-column models were trained. The multiple-column models considered both vertical and horizontal OCT images simultaneously at each CNN backbone and concatenated features from each backbone as in Fig. 1b. In this architecture, after the feature extraction layers of two single-column (vertical and horizontal) model, the multiple-column model includes a concatenation layer, which combines the features from the two single-column models, and a fully-connected layer with a batch normalization layer. In order to train efficiently, we initialized the weights of each column with each of the previously trained best performing single vertical and horizontal models. The multiple-column models were trained for 20 epochs with an initial learning rate of 0.003, batch size of 16, and stochastic gradient descent optimization.

### Evaluation of the model and experimental settings

The researchers used five-fold cross validation to tune the optimal parameters for the models using the training dataset^29^. In this method, the whole dataset was randomly divided into five subsets. Then the researchers trained a model with four of the subsets and validated the model with the remaining data (each subset included 120 cases in this study). By changing the subsets for training and validation, this process involved five iterations^30,31^. To avoid over-estimation, data from each patient were included in a single subset when possible. After finding the best parameters from the cross validation, the models were trained using the whole five-subset training dataset. The diagnostic performance of the model was evaluated with a test dataset. The area under the receiver operating characteristic curve (AUC) was calculated with each model. Experiments were implemented using Python and Keras. Training was performed on a NVIDIA GeForce GTX 1080Ti GPUs. All experiments were conducted on a 64-bit computer processor with an Intel(R) Core(TM) i7-9700 CPU @ 3.00 GHz, 8 cores.

Additional experiments were devised to evaluate the classification performance of the CNN models about side orientation. Again, the models were trained on the training dataset after five-fold cross validation and tested on the test dataset as in the previous section.

### Comparison of the model with human doctors

The test dataset was used to compare the model performance with that of retinal specialists and ophthalmology residents. For the human doctor test, a pair of vertical and horizontal OCT images was presented at the same time for classification (normal, high myopia, or other group) of the eyes. The performance of each human doctor was evaluated and compared with that of the DL models. Agreement of the five retinal specialists and four residents with the DL models was evaluated with the web-based Kappa statistics program^32^.

## Results

**Figure 2:**
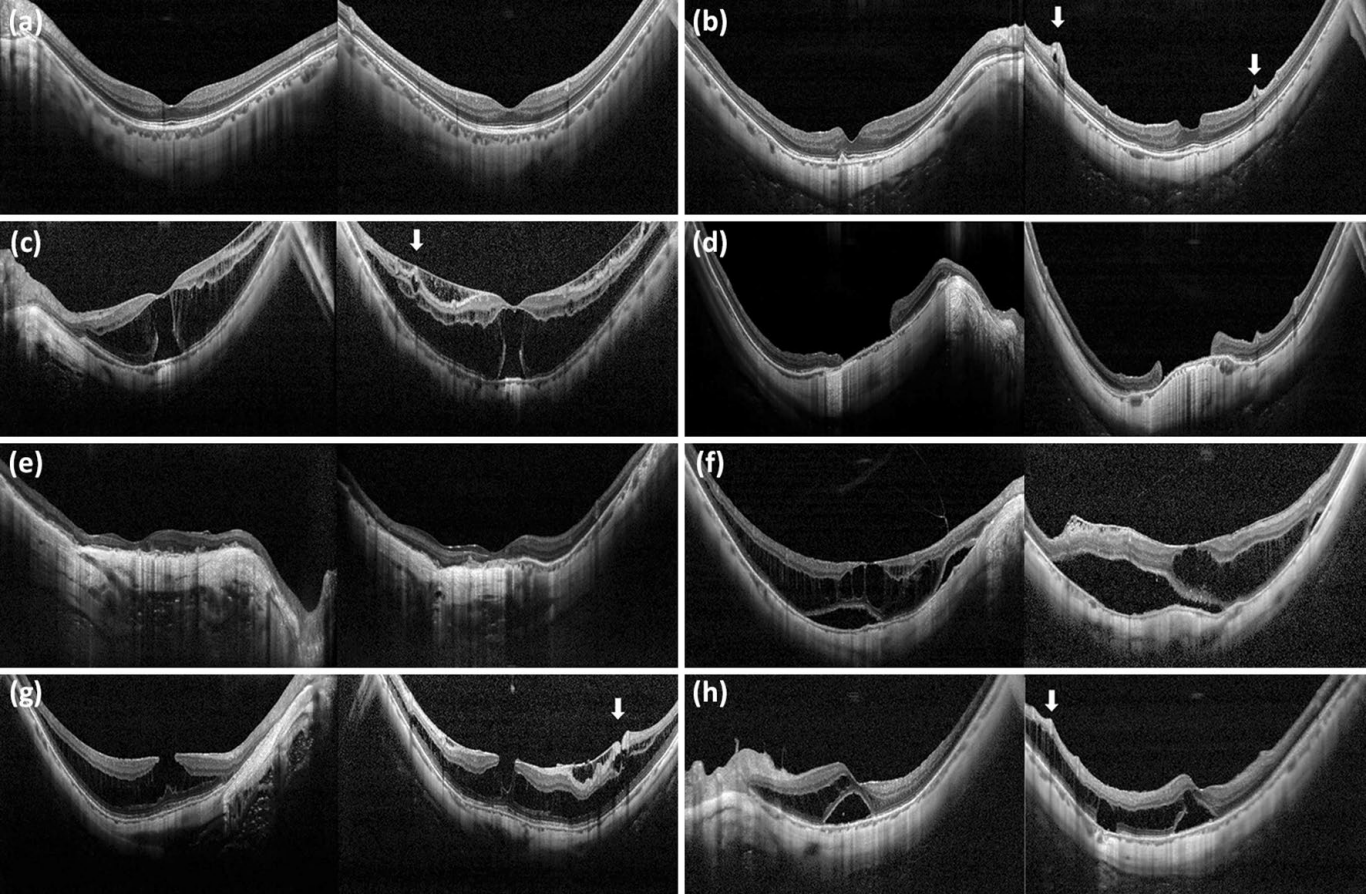
Various high myopia related features included in this study. Left column is horizontal section and right column is vertical section. (**a**) Severe curvature of posterior pole. (**b**) Paravascular retinal cysts and vascular microfolds (arrows). (**c**) Foveoschisis and impending macular hole was shown. Paravascular retinal cyst is also shown (arrow). (**d**) Macular hole and dome shape macula. (**e**) Macular chorioretinal atrophy. (**f**) Retinal detachment and retinoschisis. (**g**) Foveoshcisis and paravascular lamellar hole with retinal cysts (arrow). (**h**) Retinoschisis (arrows) with vascular microfolds and retinal detachment.

The collected 600 eyes OCT images of 436 patients were analyzed for training and validation of the proposed CNN models. Each of the three groups (normal, high myopia, and other retinal disease) included 200 eyes. Another 90 cases (30 cases for each class) were used for evaluating the trained model. Since each patient’s OCT images consisted of a pair of vertical and horizontal direction OCT images, the total training dataset consisted of 1,200 OCT scans. Various findings related to high myopia were confirmed in the OCT images included in this study (Fig. 2). The “Other” group included rhegmatogenous retinal detachment (involving the macular area), epiretinal membrane, macular hole, vitreomacular traction syndrome, diabetic retinopathy (including diabetic macular edema), age-related macular degeneration (including both dry and wet types), central serous chorioretinopathy, drusen, retinal vein occlusion, and macular telangiectasia. Demographics of the included patients are summarized in Table 1.

**Table 1:** Summary of the demographics of training, validation, and test data sets.

	Training and validation set			Test set		
No. of patients	434			58		
Age (mean ± SD)	58.85 ± 13.55			64.08 ± 10.95		
Sex, (male:female)	202:232			28:30		
No. of OCT images	1200 (600 eyes)			180 (90 eyes)		

Class	Myopia	Normal	Other	Myopia	Normal	Other
No. of patients	121	162	151	19	17	22
Age (mean ± SD)	53.7 ± 12.85	56.16 ± 13.75	66.57 ± 10.16	63.0 ± 12.21	63.23 ± 12.76	66.0 ± 7.12
Sex, (male:female)	57:64	80:82	65:86	8:11	5:12	15:7
No. of right eyes	97	103	102	17	15	15
No. of left eyes	103	97	98	13	15	15
Axial length (mean ± SD)	29.25 ± 2.27	23.75 ± 0.96	23.46 ± 0.89	29.23 ± 1.72	23.53 ± 1.13	23.72 ± 0.91

Class myopia included eyes with axial length of 26.5 mm or more.

Class normal and other included eyes with axial length between 21.5 mm and 26.0 mm.

### Cross validation results on training dataset

Table 2 denotes the five-fold cross validation results of the single- and multiple-column models. The VGG 16- and ResNet 50-based single-column CNN models revealed a greater than 0.97 average area under the receiver operating characteristic curve (AUC) for both vertical and horizontal images. In the multiple-column CNN models, when initializing with the ImageNet-pretrained models, training of the VGG 16 and ResNet 50 networks was difficult. Inception V3 was better than the other networks, but the performance was relatively poor. The authors then tried to initialize the models with the pretrained single-column models shown in Table 2. ResNet 50 models with single-column initialization showed perfect classification performance from the five-fold cross validation on the training dataset.

**Table 2:** Five-fold cross validation results for each of the single- and multiple-column models.

CNN backbone	Micro-average AUC of the single-column models		Micro-average AUC of the multiple-column models	
			Initialization with ImageNet-pretrained models	Initialization with the pretrained single-column models
VGG 16	Vertical	0.9859 ± 0.00 (0.9826–0.9906)	0.5801 ± 0.07 (0.5189–0.6409)	0.6827 ± 0.17 (0.5307–0.8341)
	Horizontal	0.9873 ± 0.01 (0.9811–0.9934)		
Resnet 50	Vertical	0.9746 ± 0.01 (0.9646–0.9850)	0.5545 ± 0.08 (0.4829–0.6261)	1.0000 ± 0.00 (1.0–1.0)
	Horizontal	0.9844 ± 0.01 (0.9796–0.9896)		
Inception V3	Vertical	0.8967 ± 0.04 (0.8625–0.9310)	0.8048 ± 0.07 (0.7455–0.8648)	0.9170 ± 0.03 (0.8886–0.9453)
	Horizontal	0.9188 ± 0.04 (0.8809–0.9568)		

*CNN* convolutional neural network, *AUC* area under the receiver operating characteristic curve.

Data are mean ± standard deviation (95% confidence interval).

### Results on the test dataset

**Figure 3:**
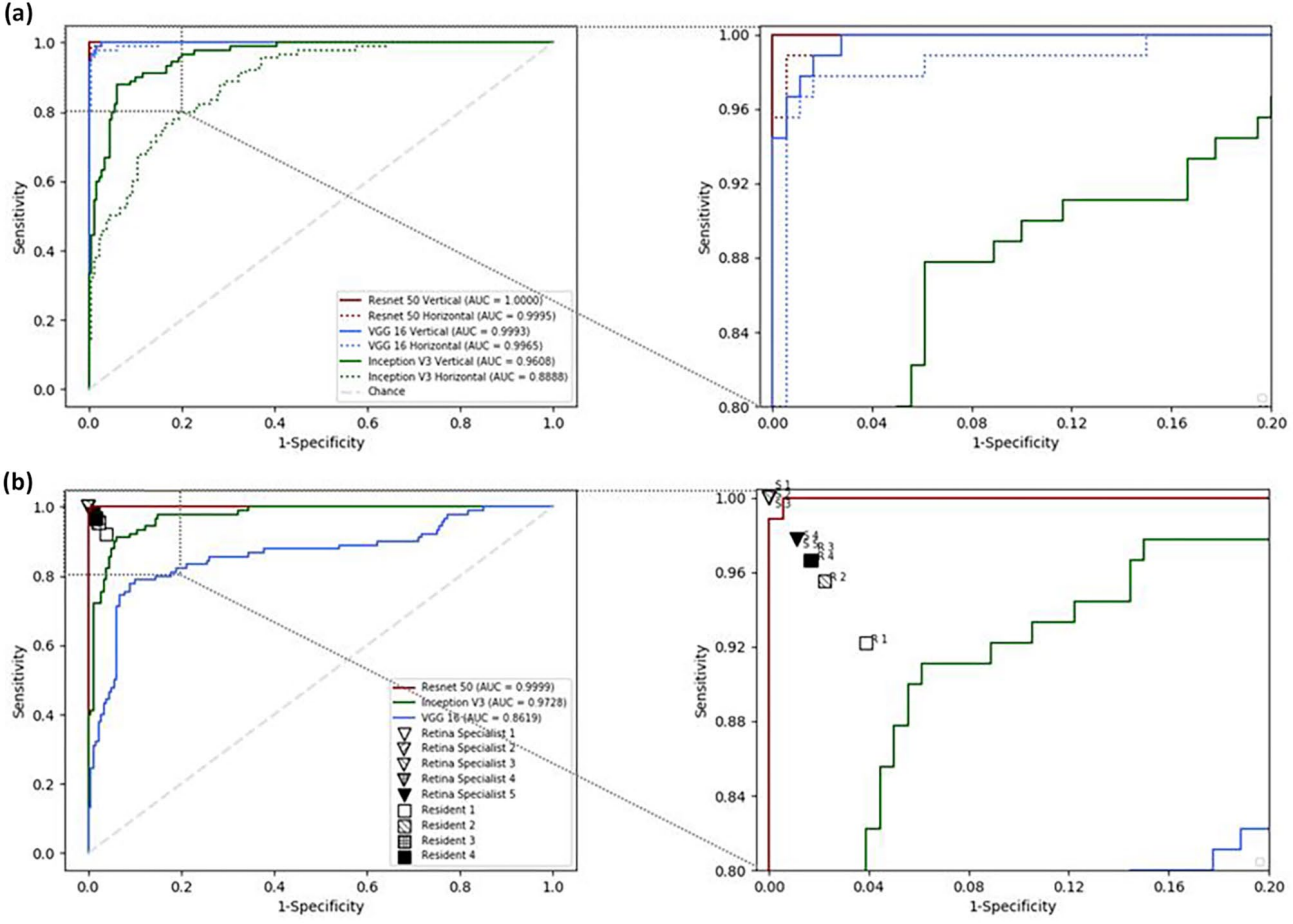
Comparison of the performance of the deep learning models with that of human doctors. (**a**) The receiver operating characteristic curves of three deep learning models of a single-column model. ResNet 50 demonstrate the best diagnostic performance. (**b**) The receiver operating characteristic curves of three deep learning models of the multiple-column model and the diagnostic performance of human doctors. ResNet 50 show the best diagnostic performance among the three deep learning models and had comparable performance to that of the retinal specialists.

To evaluate the performance of the trained models, a test dataset was analyzed as above. The same preprocessing was performed on the test dataset as on the training dataset without the data augmentation. Table 3 shows the test results of the single- and multiple-column models. The ResNet 50 single-column model presented the highest classification accuracies on the test dataset (100% and 98.89% for vertical and horizontal models, respectively). For the multiple-column model, which considers vertical and horizontal OCT images at the same time for classification of the case, the ResNet 50 showed the highest 100% classification results on the test dataset. Figure 3 shows the AUCs of DL models as 0.99, 0.97, and 0.86 for ResNet 50, Inception V3, and VGG 16, respectively.

**Table 3:** Absolute agreement and intergrader agreement of the deep learning models, retinal specialists, and resident ophthalmologists.

CNN backbone	Absolute agreement of single-column model		Absolute agreement of multiple-column model	Cohen Kappa (95% confidence interval)
VGG 16	Vertical	97.78% (88/90)	72.22% (65/90)	0.52 (0.38–0.66)
	Horizonal	96.67% (87/90)		
Resnet 50	Vertical	100.00% (90/90)	100.00% (90/90)	1.0 (1.0–1.0)
	Horizonal	98.89% (89/90)		
Inception V3	Vertical	88.89% (80/90)	90.00% (81/90)	0.85 (0.76–0.94)
	Horizonal	87.78% (79/90)		

Human doctors	Absolute agreement (range)			Cohen Kappa (range)
Resident ophthalmologists (range)	95.28 ± 2.1% (92.22–96.67%)			0.93 ± 0.03 (0.88–0.95)
Retinal specialists (range)	99.11 ± 1.22% (97.78–100%)			0.99 ± 0.02 (0.97–1.0)

*CNN* convolutional neural network.

(No. of correct diagnosis/no. of test set).

Results of human doctor is given as mean ± standard deviation (four residents and five retinal specialists).

The Cohen κ statistic was evaluated as follows: 0.21 to 0.40 indicated fair agreement; 0.41 to 0.60, moderate agreement; 0.61 to 0.80, substantial agreement; and 0.81 to 1.0, near-perfect agreement.

**Figure 4:**
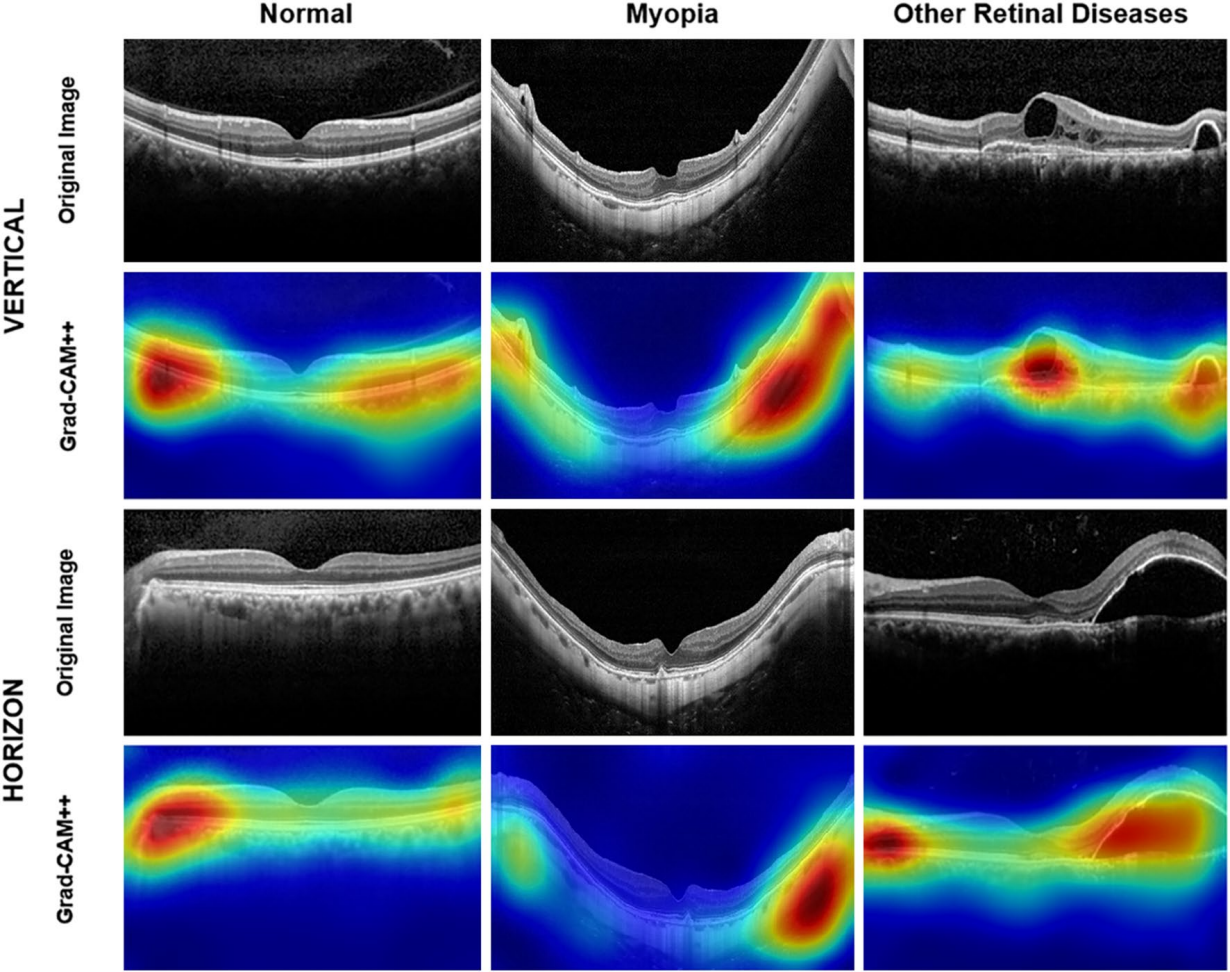
Visual explanations generated by Grad-CAM +  + on OCT scans. The Grad-CAM +  + image show that the deep learning model accurately identified the differentiation points.

**Figure 5:**
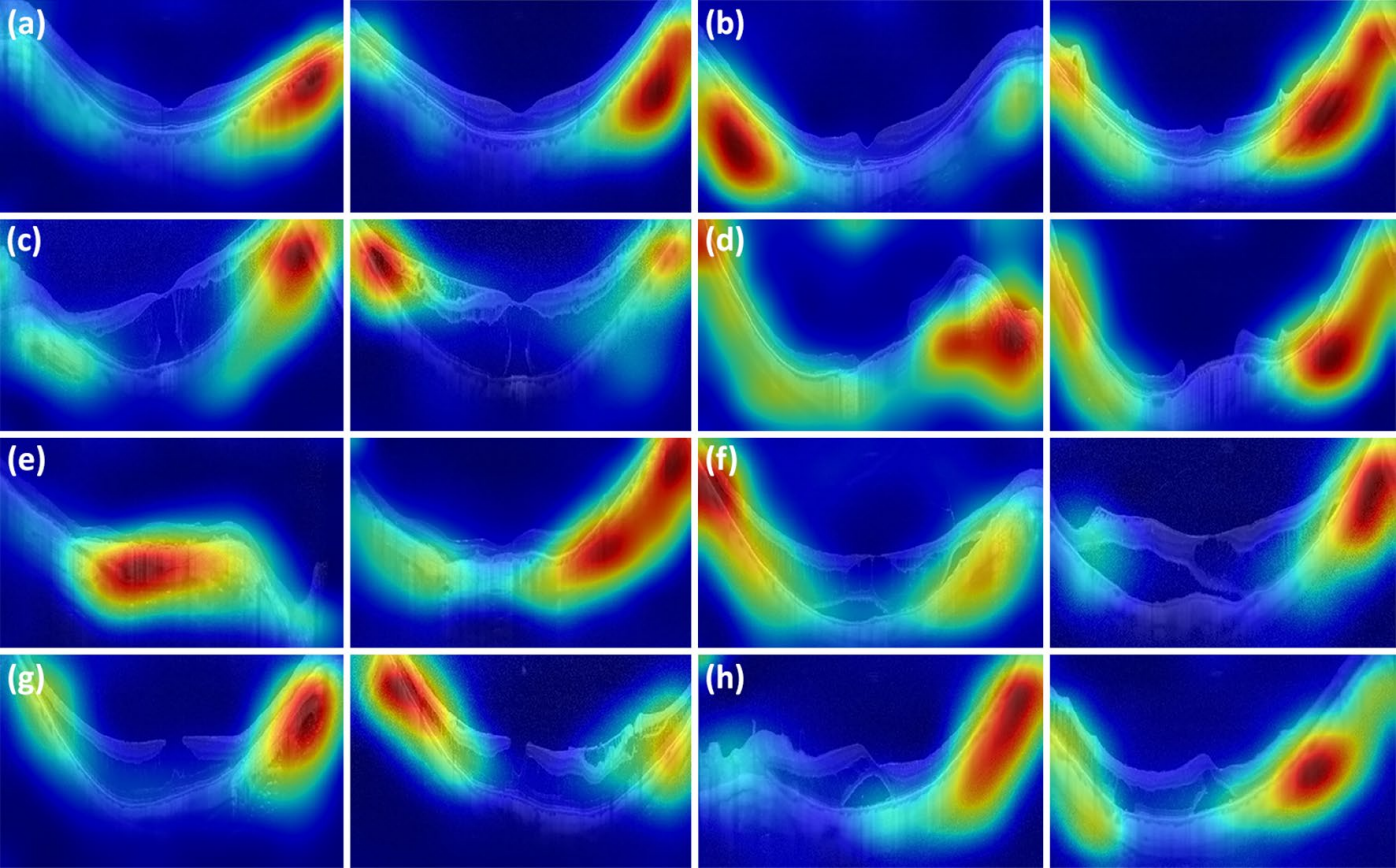
Visual explanations generated by Grad-CAM +  + on OCT scans of myopia class. The Grad-CAM +  + image show that the deep learning model identified some of the characteristic features of high myopia. The deep learning model accurately identified severe curvature of high myopic eye for all of the OCT images. The model also identified vascular microfolds (**b**), peripapillary artrophy (**c**, **d** and **e**), chorioretinal macular atrophy (**e**), Retinal cysts and paravascular lamellar hole (**g**) and retinoschisis (**g** and **h**).

Gradient-weighted class activation mapping (Grad-CAM + +) is a visualization tool that helps interpret the classification results on each input image by heatmap (red areas denote the locations where the model looked for the predicted class)^33,34^. The visual explanations generated by Grad-CAM +  + on input OCT scans are shown in Figs. 4 and 5. The Grad-CAM +  + image showed that the DL model accurately identified the parts of the differentiation point among 3 classes (Fig. 4). It was also confirmed that some characteristic parts of the various high myopia features included in this study were detected by the DL models from the Grad-CAM +  + image (Fig. 5).

### Classification performances of left vs. right and vertical vs. horizontal OCT images

Additionally, experiments were performed to see if CNN models could classify the OCT images as left or right eye images and vertical or horizontal images. The ResNet 50 model showed 100% classification performance for both tasks. VGG 16 and Inception V3 models showed 98.89% and 93.33% accuracy rates in left–right classification, respectively, and 98.89% and 88.89% accuracy rates in vertical-horizontal classification. Since the left–right classification task is only possible on horizontal images by location of optic discs, only horizontal images were included for the task.

### Comparison of performances between the CNN model and human doctors

To compare the performance of the model with that of human doctors, nine human doctors participated in the evaluation with the test dataset, which was the same set as used to evaluate the DL model. The nine doctors consisted of four residents and five retinal specialists. Table 3 shows the test results of the DL models and human doctors. The four residents had a 95.28% correct answer rate, and the five retinal specialists had a 99.11% correct answer rate. Both the VGG 16 and the Inception V3 multiple-column model were less accurate than the human doctors. However, the ResNet 50 model showed comparable performance to the retinal specialists, with the highest kappa value among the 3 DL models. The model outperformed all four ophthalmologists-in-training and two of the five retinal specialists. Comparison of receiver operating characteristic curves of the three DL models with the performance of human doctors is shown in Fig. 3b.

## Discussion

This study demonstrated that a DL model using OCT images could distinguish accurately high myopia from normal and other macular diseases. Also, the performance of the model was comparable to that of retinal specialists. In this study, patients with high myopia and normal vision were divided based on axial length, and the DL model was trained based on the dataset created by retinal specialists annotating normal and other macular diseases for the normal axial length image. With this training, the DL model achieved a very high performance regarding correct answers for the test dataset configured separately from the training dataset. This is the first attempt to diagnose high myopia with OCT using a DL algorithm.

High myopia is one of the major causes of visual impairment worldwide, is accompanied by many ophthalmic complications, and has a high prevalence rate, especially in Asians^1,2,35–37^. However, since axial length is not usually measured in ordinary ophthalmic examinations, except for cases where axial length is measured as in pre-cataract surgery, it might be useful to be able to confirm high myopia through other examinations. As the population who has undergone refractive surgery such as laser vision correction or cataract surgery increases, the situation in which it is difficult to confirm high myopia only with refraction test is expected to increase^38–40^. Therefore, if high myopia is sufficiently confirmed by OCT alone in patients who have already taken OCT for retinal disease, additional cost and time for axial length measurement are not required. In this regard, we think that OCT with DL algorithm in our study will be clinically useful in such cases, especially for non-retinal specialists. In addition, through this study, it was confirmed that the DL model can distinguish and detect some of the characteristic findings of OCT found in high myopia. We also expect it will serve as a basis for the development of more advanced DL model which can distinguish and diagnose the various macular disease with OCT images.

Interestingly, a method capable of estimating or calculating the axial length using only OCT rather than A-scan or partial coherence interferometry has been introduced^41–44^. In those study, with the sequential anterior and posterior OCT systems or the whole-eye OCT system (simultaneous anterior and posterior OCT system), the anterior segment and posterior segment images were acquired in an OCT scan. With this technique, it is possible to estimate the axial length from OCT images. This whole-eye OCT system seems to be very promising, however, the system has not yet been commercialized, not widely distributed, and has been of limited use usually in a research so far.

The various OCT findings of high myopia identified in this study were reported in previous studies, and some of the characteristic findings were detected in the DL model (Figs. 2 and 5). Paravascular retinal cysts are small hollow spaces around large retinal vessels and are often identified by OCT examination of high myopic eyes^45,46^. Vascular microfolds, which occur due to inflexibility of retinal vessels, are also common OCT features of high myopia. There were reports that the frequency of retinoschisis was high when the vascular microfolds observed together with paravascular retinal cysts^9,45^. Paravascular lamellar holes are found around paravascular retinal cysts, and paravascular retinoschisis is also frequently observed together^45^. Myopic tractional maculopathy and myopic foveoschisis are also not uncommon findings in high myopic eyes, and it is thought that it may be caused by the poorly stretched internal limiting membrane not keeping pace with the progression of posterior staphyloma^47–50^. Choroidal neovascular membrane is an important complication of high myopia that can cause visual loss^3^. Myopic choroidal neovascularization shows subretinal hyperreflective material in an active state, with or without subretinal fluid^8^. Thereafter, it progresses to the scar stage and the atrophic stage. Macular hole and posterior retinal detachment in highly myopic eyes may occur simultaneously or separately and may cause visual loss which need surgical intervention^10,51,52^.

According to a recent study, the performance of the DL model was better when all three of the annotations of the retinal specialists for fundus photography were the same than when only two of the annotations were the same^15^. In other studies, the overall performance of the DL model improved after correct labeling^16^. Therefore, accurate annotation is very important in training DL models. In this study, annotation was performed for high myopia based on the objective value of an axial length, and the DL model trained with the data set constructed based on this showed excellent performance. This suggests that objective and accurate annotation of OCT images is important for the DL model with OCT images. Interestingly, DL models generated with three CNN architectures were validated and tested, and each showed unique diagnostic performance. This suggests that certain CNN architecture is more suitable for a specific situation such as a limited amount of data or certain purpose. Further studies are needed to determine the algorithms for finding appropriate CNN architectures.

This study’s model architecture has technical advantages. Despite the relatively small amount of training data, there was high performance among three classes due to various data augmentation techniques such as brightness adjustment and random shift cropping. The right-eye OCT images were flipped to the left for consistent optic disc location and better classification results. Moreover, there was 100% classification performance from both cross validation and tests when using the multiple-column CNN models. It is more natural to use the multiple-column models to feed both vertical and horizontal images as ophthalmologists read both images at the same time for diagnosis. Although the single-column models showed high performance with ImageNet pretrained model initialization, it was difficult to train the multiple-column models. This is because the amount of training data was not enough to train the more complex multiple-column models with twice as many parameters to train as the single-column models. Consequently, the multiple-column models were initialized with the pretrained single-column models to overcome this problem.

The results of additional experiments about classification performance of vertical vs. horizontal OCT images demonstrated the feasibility of a fully automatic framework to read OCT images for high myopia and other retinal diseases. When a pair of OCT images of an eye is input into the framework, it can classify them automatically between vertical and horizontal images and then feed each one into the corresponding column of the multiple-column CNN model to create classification output of the eye. This whole process is depicted in supplementary Fig. S1. Because there were high classification performances for vertical versus horizontal classification, the fully automatic framework could be implemented in a clinical setting.

This study had limitations. First, it was a retrospective study, targeting patients whose axial length was measured for cataract surgery. Because of this, it is possible that a consecutive and mixed series of patient groups was included. In addition, the relatively young age group who did not undergo cataract surgery was not included. Second, the DL model needs to be validated with an external dataset. In addition, it is necessary to verify the validity of images acquired from OCT equipment other than that used in this study. The diagnostic performance of this model for external datasets or other OCT equipment images is likely to be lower than the results of this study. Third, in this study, diagnosis was divided into three categories: high myopia, normal, and other macular diseases; additional subdivided categories are needed in actual clinical situations. In particular, other macular disease comprises a wide variety of diseases, and it is important to divide and annotate these groups accurately. Fourth, considering the various OCT features of the myopic eye, the number of cases may be rather small to include the diversity. However, as mentioned above, relatively diverse myopic features are included in the OCT images of this study, and some of the non-included findings may be related to the specificity of the OCT image obtained before cataract surgery. Lastly, the DL model of this study was not designed to differentiate between eyes with just high myopia and eyes with high myopia and other retinal diseases, so it was impossible to evaluate this function. However, this distinction can be clinically useful and should be applied in the future research and development. It is thought that these issues must be overcome with the additional research or development with more diverse OCT images and external datasets.

Despite some limitations, the DL model of this study, which is comparable to that of retinal specialists and showed reliable performance, is considered a very high possibility for clinical utility. The high accuracy and performance of the DL model can be of great help to general ophthalmologists or general practitioners in screening and diagnosis on OCT images. In addition, as already demonstrated, the usefulness of Grad-CAM +  + images in other fundus image-related DL algorithm studies and OCT-related DL algorithm studies, this study showed that the Grad-CAM +  + image can provide a clue for interpretation of the result of a DL model^15,21^ (Figs. 4 and 5). This Grad-CAM +  + image is thought to be useful to ophthalmologists or specialists by quickly guiding lesions that are the basis for diagnosis. A more accurate diagnostic approach will be possible if the DL model with Grad-CAM +  + is correlated with clinical information such as vision and intraocular pressure.

## Conclusion

In this study, the deep learning model using OCT images demonstrated reliable diagnostic performance for high myopia and comparable performance to that of retinal specialists.

## Supplementary Information

Supplementary Figure S1.

## Data Availability

The data used and/or analyzed during the current study are available from the corresponding author upon request.
