# Supplementary material for: Deep learning models for screening of high myopia using optical coherence tomography
Source: Sci Rep. 2021 Nov 4;11:21663. doi: 10.1038/s41598-021-00622-x (PMC8568935; doi:10.1038/s41598-021-00622-x)
Supplement: Supplementary file 1 — Supplementary Figure S1. [file 41598_2021_622_MOESM1_ESM.docx]

**Supplementary Information**

**Deep learning models for screening of high myopia using optical coherence tomography**

Kyung Jun Choi^1^, Jung Eun Choi^2^, Hyeon Cheol Roh^3^, Jun Soo Eun^4^, Jong Min Kim^5^, Yong Kyun Shin^1^, Min Chae Kang^1^, Joon Kyo Chung^1^, Chae Yeon Lee^1^, Dong Young Lee^1^, Se Woong Kang^1^, *Baek Hwan Cho^2,6^, *Sang Jin Kim^1^

Main authors for correspondence:

These authors contributed equally as corresponding authors: Sang Jin Kim, Baek Hwan Cho

**Address correspondence and reprint requests to**

Sang Jin Kim M.D., Ph.D.,

Department of Ophthalmology, Samsung Medical Center, Sungkyunkwan University School of Medicine, #81 Irwon-ro, Gangnam-gu, Seoul, 06351, Republic of Korea

email: [sangjin.kim.md@gmail.com](mailto:sangjin.kim.md@gmail.com)

Baek Hwan Cho Ph.D.,

Medical AI Research Center, Samsung Medical Center, #81 Irwon-ro, Gangnam-gu, Seoul 06351, Republic of Korea

Department of Medical Device Management and Research, SAIHST, Sungkyunkwan University, Seoul 06351, Republic of Korea

email: baekhwan.cho@samsung.com


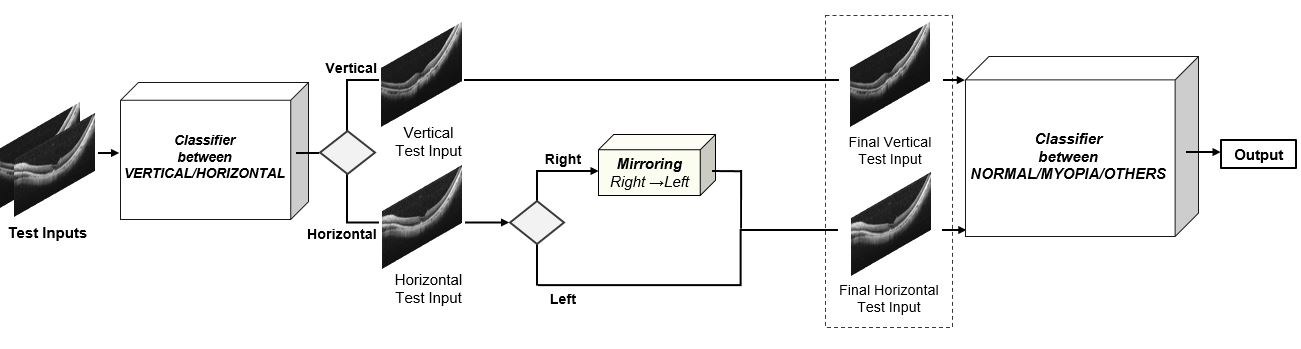


**Supplementary Figure S1.** Fully automatic test framework. A pair of OCT images of an eye is automatically classified between vertical and horizontal images, and then each one is fed into the corresponding column of the multiple-column CNN model to classify the eye.
